# Bioinformatics Identification of Ferroptosis-Associated Biomarkers and Therapeutic Compounds in Psoriasis

**DOI:** 10.1155/2022/3818216

**Published:** 2022-10-12

**Authors:** Jingyi Mao, Xin Ma

**Affiliations:** ^1^Department of Dermatology, Shuguang Hospital Affiliated to Shanghai University of Traditional Chinese Medicine, Shanghai, China 200000; ^2^Shanghai Skin Disease Hospital, Tongji University School of Medicine, Shanghai, China 200000

## Abstract

**Purpose:**

Psoriasis is closely linked to ferroptosis. This study aimed to identify potential ferroptosis-associated genes in psoriasis using bioinformatics.

**Methods:**

Data from the GSE30999 dataset was downloaded from the Gene Expression Omnibus (GEO), and the ferroptosis-associated genes were retrieved from FerrDb. The differentially expressed ferroptosis-associated genes were identified using Venn diagrams. Subsequently, a network of protein-protein interactions (PPIs) between psoriasis targets and ferroptosis-associated genes was constructed based on the STRING database and analyzed by Cytoscape software. The Metascape portal conducted Gene Ontology (GO) and Kyoto Encyclopedia of Genes and Genomes (KEGG) pathway enrichment analyses. Moreover, the expression of ferroptosis-related genes was verified in the GSE13355 dataset. Finally, the verified genes were used to predict the therapeutic drugs for psoriasis using the DGIdb/CMap database. SwissDock was used to examine ligand docking, and UCSF Chimera displayed the results visually.

**Results:**

Among 85 pairs of psoriasis lesion (LS) and no-lesion (NL) samples from patients, 19 ferroptosis-associated genes were found to be differentially expressed (3 upregulated genes and 16 downregulated genes). Based on the PPI results, these ferroptosis-associated genes interact with each other. The GO and KEGG enrichment analysis of differentially expressed ferroptosis-related genes indicated several enriched terms related to the oxidative stress response. The GSE13355 dataset verified the results of the bioinformatics analysis obtained from the GSE30999 dataset regarding SLC7A5, SLC7A11, and CHAC1. Psoriasis-related compounds corresponding to SLC7A5 and SLC7A11 were also identified, including Melphalan, Quisqualate, Riluzole, and Sulfasalazine.

**Conclusion:**

We identified 3 differentially expressed ferroptosis-related genes through bioinformatics analysis. SLC7A5, SLC7A11, and CHAC1 may affect the development of psoriasis by regulating ferroptosis. These results open new avenues in understanding the treatment of psoriasis.

## 1. Introduction

Psoriasis is a systemic inflammatory disease related to the increased risk of comorbidities such as inflammatory arthritis, Crohn's disease, malignant tumor, and cardiovascular disease [1]. Carriage of HLA-Cw6, as well as environmental triggers (such as streptococcal infection, stress, smoking, obesity, and alcohol consumption) are major determinants of disease expression, and the abnormal proliferation and differentiation of keratinocytes caused by persistent inflammation is the pathological feature of psoriasis [2]. Psoriasis cannot be cured, and patients need long-term treatment. Biological agents, which can effectively inhibit TNF- *α*, P40 IL-12/23, IL-17, and P19 IL-23, but have side effects, drug resistance, and other problems associated [3].

Thus, it is essential to understand the biological functions involved in the pathogenesis to overcome drug resistance and find novel and effective therapeutic targets for psoriasis.

Ferroptosis is a newly discovered form of cell death mediated by lipid peroxidation and iron overload. It differs from other types of cell death in terms of its morphology, biology, and genetics [4, 5]. Studies have shown that ferroptosis can trigger and amplify inflammatory responses [6, 7], and ferroptosis inhibitor has an anti-inflammatory effect in experimental models of several diseases [8, 9]. A recent study found that ferroptosis-associated cell death was activated in psoriasis lesions. A similar ferroptosis tendency was also observed in human primary keratinocytes treated with erastin and imiquimod- (IMQ-) induced psoriasis models. Ferrostatin-1 (Fer-1), an effective lipid peroxidation inhibitor, can inhibit the changes related to ferroptosis in erastin-treated keratinocytes and alleviate psoriasis-like dermatitis in IMQ induced model. Moreover, Fer-1 blocks inflammatory responses *in vitro* and *in vivo*, reducing the production of cytokines such as IL-1*α*, IL-1*β*, IL-6, IL-17, IL-22, IL-23, and TNF-*α*. This study revealed the expression pattern of ferrostatin, a specific molecule involved in ferroptosis, that enhances the inflammatory reactions in psoriasis [10].

The GSE30999 dataset, generated by Suárez-Fariñas M et al., obtained differentially expressed genes (DEGs) between psoriasis lesions and control skin tissues from patients with moderate to severe psoriasis vulgaris [11]. The result showed that when defined by >2-fold change (FCH) and false discovery rate (FDR) <0.05, 2725 genes were differentially expressed in the two groups. Here, we studied the dataset again from other perspectives. By defining adjusted *P-*value <0.05 and |log2FC| > 1.5, we explored the differentially expressed ferroptosis-associated genes in psoriasis by analyzing the GSE30999 dataset in the GEO database. The differentially expressed ferroptosis-associated genes were analyzed by Protein-Protein Interaction (PPI), correlation analysis, Gene Ontology (GO), and Kyoto Encyclopedia of Genes and Genomes (KEGG) pathway enrichment analysis. Then, the expression level of the differentially expressed ferroptosis-related genes was further verified in the GSE13355 dataset. Finally, the DGIdb/CMap database predicted therapeutic drugs, and molecular docking was carried out by SwissDock/UCSF Chimera software.

## 2. Materials and Methods

### 2.1. Ferroptosis-Associated Genes Datasets and Microarray Data

A total of 359 ferroptosis-associated genes were obtained from the FerrDb database (http://www.zhounan.org/ferrdb/) [12]. The mRNA expression profile dataset of GSE30999 was downloaded from the GEO database (http://www.ncbi.nlm.nih.gov/geo/). GSE30999 was in the GPL570 platform (Affymetrix human genome U133 Plus 2.0 array), which contained 81 pairs of psoriasis lesions (LS) and no-lesion (NL) samples from patients with moderate to severe psoriasis, and 4 LS and 4 NL unpaired. RMA (Robust Multiarray Average) was used for data preprocessing, including background adjustment, quantile method standardization, and expression calculation. If a certain probe could not match a specific gene symbol, it would be excluded. And if multiple probes correspond to the same gene, the median expression of each probe was taken as the gene expression.

### 2.2. Differential Expression Analysis of Ferroptosis-Associated Genes

DEGs were identified from the GSE30999 dataset using the “limma” package of R software (version 1.2.5001). Genes with an adjusted *P-*value <0.05 and |log2FC| > 1.5 were regarded as DEGs. Subsequently, the intersection of ferroptosis-associated genes and candidate genes with DEGs, were obtained. “Heatmap” and “ggplot2” software packages of R software were used to generate volcano maps, heat maps, and block diagrams.

### 2.3. PPI and Correlation Analysis of the Differentially Expressed Ferroptosis-Associated Genes

The STRING database (https://string-db.org/) and Cytoscape software (version 3.8.1) were used for PPI analysis of the differentially expressed ferroptosis-related genes. The correlation analysis of differentially expressed ferroptosis-associated genes was carried out using the spearman correlation in the “corrplot” software package of R software.

### 2.4. Enrichment Analysis of Ferroptosis-Associated Genes by GO and KEGG Pathway

Metascape was used to conduct a functional analysis of the obtained DEGs (https://metascape.org/gp/index.html#/main/step1) [13]. The ferroptosis-associated genes were loaded into Metascape, and terms with *P* < 0.01 and |log2FC| > 1.5 were defined as significant. The chord diagram and enrichment analysis were conducted using the “GO plot” in R software. The GO analysis consisted of cellular components (CC), biological processes (BP), and molecular functions (MF).

### 2.5. Validation of Gene Expression Associated with Ferroptosis

The GSE13355 dataset was downloaded from the GEO database, which contained samples from 58 psoriasis patients with skin lesions and 64 control samples from healthy people. RMA was used for data preprocessing, including background adjustment, quantile method standardization, and expression calculation. The exclusion criteria of probes was the same as those in 2.1. The differentially expressed genes were screened using the R “limma” software package (version 1.2.5001) according to the adjusted *P-*value <0.05, *P-*value <0.05, and |log2FC| > 1.5. Finally, the intersection of ferroptosis-associated genes, GSE30999, and GSE13355 differential genes, were obtained.

### 2.6. Molecular Docking Analysis

Dgibd database [14] (https://dgidb.org/) and CMAP database [15] (https://clue.io/) were used to examine potential drugs targeting ferroptosis-related genes. Appropriate target proteins were screened to understand the docking mode of drug targets based on the following conditions: (1) obtained from *Homo sapiens*, (2) the resolution must be less than 3.5 Å, (3) the sequence of conformation should be nearly complete, and there must be small molecule ligand information in the structural complex, (4) if two or more structures were available, the structure with the best solution was selected. 3D structures of SLC7A5 and SLC7A11 were downloaded from the RCSB Protein Data Bank (RCSB-PDB; http://www.rcsb.org) [16]. Compound structures of Melphalan, Quisqualate, Riluzole, and Sulfasalazine were downloaded from the ZINC database (https://zinc.docking.org/substances/home/) [17]. Original ligands and water molecules of the target proteins were deleted in the structural formula, and ligands were targeted through the UCSF chimera software (version 1.16). SwissDock (http://www.swissdock.ch/docking)was used to perform a docking simulation to verify the credibility of the ferroptosis-related genes [18]. Finally, UCSF Chimera was used to evaluate the possible binding mode and generate the interactive data visualization from SwissDock. Fullfitness energy is an estimate of docking accuracy; its low score indicates good docking effect. Negative energy indicates that the receptor and ligand can bind spontaneously, and energy less than -5 kcal/mol would mean a good binding activity between them.

### 2.7. Statistical Analysis

The data are shown as mean ± standard deviation (SD). All the statistical analyses were performed in R software, and a *P*-value <0.05 was considered statistically significant. The unpaired student's *t*-test was employed to determine *P* values and adjusted *P* values in the DEG analysis, where *P* values were adjusted by false discovery rates (FDR). The differences between two groups were analyzed using the Wilcox test or *t*-test according to the data distribution characteristics.

## 3. Results

### 3.1. Differential Expressions of Ferroptosis Signatures in Psoriasis

**Figure 1:**
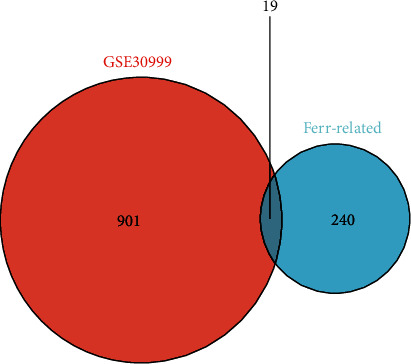
Differential expressions of ferroptosis-related genes. Venn diagram showing the intersection between differentially expressed genes and ferroptosis-related genes. The red color represents differentially expressed genes in the GSE30999 dataset, and the blue color represents ferroptosis-related genes.

The GEO datasets contained 81 paired samples, 4 LS, and 4 NL unpaired samples (Table 1). A total of 54675 probe sets (23520 unique known genes) were collected. The differential expression of LS and NL was analyzed through the “limma” package of R software. |log2fc| = 1.5, *P* value <0.05 and *P* value after correction <0.05 was considered statistically significant. A total of 920 DEGs were screened. A dataset containing 359 genes was obtained from the ferroptosis database (FerrDb). The DEGs obtained from the GSE30999 dataset intersected with the ferroptosis genes. The Venn diagram showed 19 differentially expressed genes related to ferroptosis (Figure 1).

### 3.2. Analyzing Differential Expressions of Ferroptosis-Associated Genes

**Figure 2:**
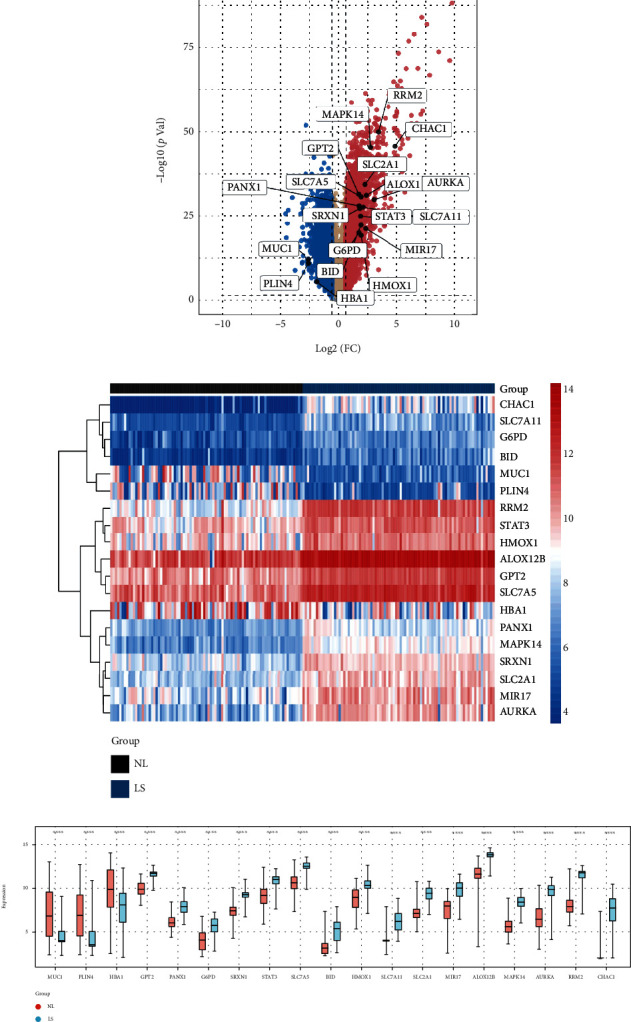
Analyzing differential expressions of ferroptosis-associated genes. (a) Volcano plot of differentially expressed RNA between psoriasis lesions and control samples in the GSE30999 dataset, highlighting the differentially expressed ferroptosis-associated genes. The values marked with dotted lines represented |log2fc| = 1.5. The top shows the number of upregulated (red) and downregulated (blue) genes. (b) Heatmap of differentially expressed ferroptosis-associated RNA in the GSE30999 dataset. Red represents high expression, and blue represents low expression. The Ward's minimum-variance hierarchical clustering method was used. (c) The box diagram showing 19 differentially expressed ferroptosis-associated genes in psoriasis and control samples. Gene names were sorted by log2FC value from large to small. The Wilcox test was used. ^∗∗∗∗^- *P* value <0.0001 compared to control.

The volcano and heat maps showed 19 differentially expressed ferroptosis-associated genes between psoriasis and control samples (Figures 2(a) and 2(b)). In addition, the expression pattern of differentially expressed ferroptosis-associated genes is shown the block diagrams (Figure 2(c)). Three of the 19 ferroptosis-associated genes, MUC1, PLIN4, and HbA1, were downregulated. The top five upregulated genes included CHAC1, RRM2, AURKA, MAPK14, and ALOX12B (Table 2).

### 3.3. PPI and Correlation Analysis of the Differentially Expressed Ferroptosis-Associated Genes

**Figure 3:**
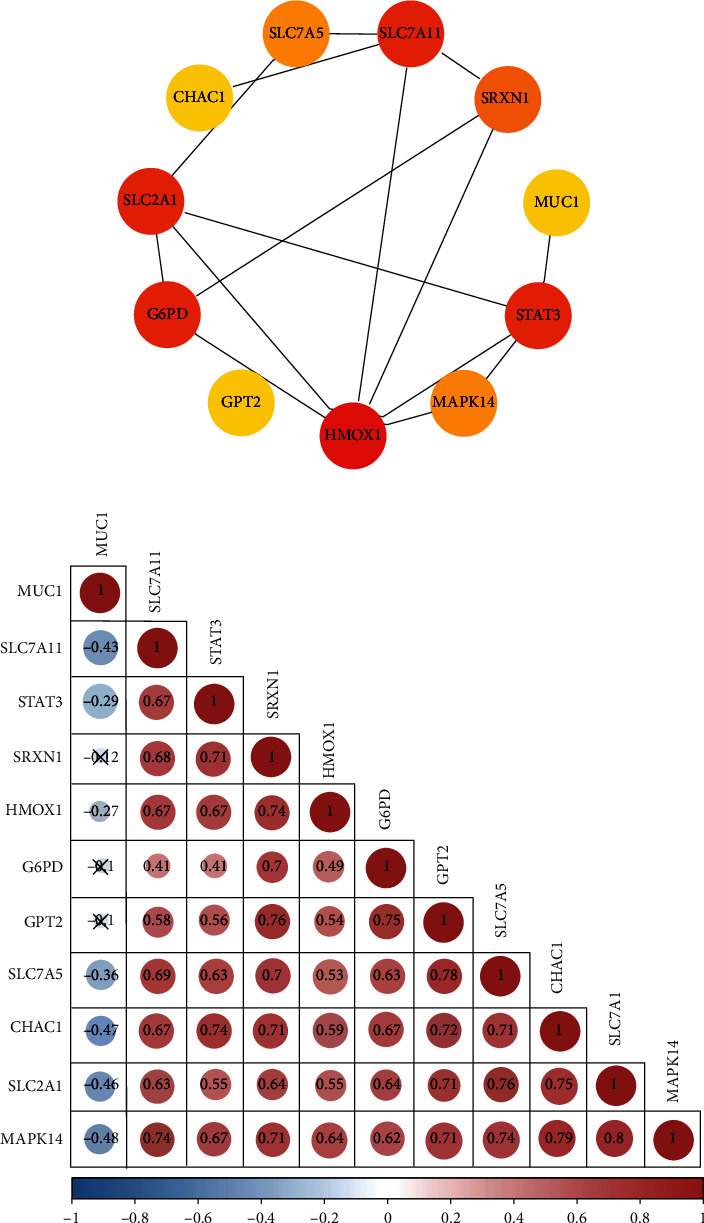
PPI and correlation analysis of the differentially expressed ferroptosis-associated genes. (a) A PPI network showing the interaction between differentially expressed ferroptosis-associated genes. The node color depth represented the degree value. (b) Correlation analysis shows a correlation between the 11 differentially expressed ferroptosis-associated genes in the GSE30999 dataset.

To determine the interaction between differentially expressed ferroptosis-associated genes, we analyzed 19 differential genes and obtained a PPI network with 18 nodes and 17 edges. Among them, 11 genes, including MUC1, GPT2, G6PD, SRXN1, STAT3, SLC7A5, HMOX1, SLC7A11, SLC7A1, MAPK14, and CHAC1, formed molecular networks. The other eight were neither related to other genes nor formed molecular networks. The network was set to the default cutoff point in the STRING database. Nodes represented genes, and edges indicated interactions between genes. The PPI network diagram showed the interaction and degree value of 11 ferroptosis-associated genes (Figure 3(a)). Subsequently, a correlation analysis was performed to probe the association between the expression of these ferroptosis-associated genes (Figure 3(b)).

### 3.4. Enrichment Analysis of Ferroptosis-Associated Genes by GO and KEGG Pathway

**Figure 4:**
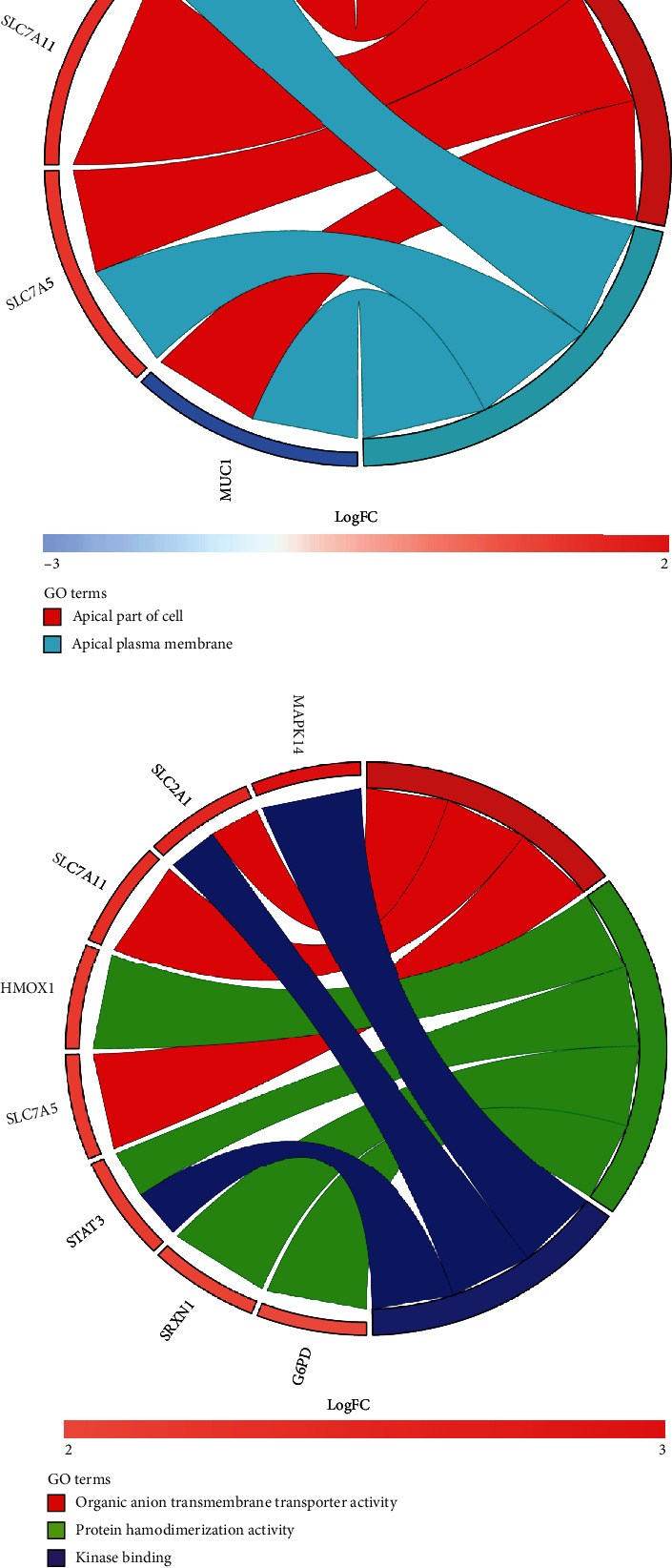
Analysis of differentially expressed genes with GO and KEGG pathways. The chord diagram shows the enrichment analysis of genes. (a) The biological process of GO analysis. (b) The cellular components of GO analysis. (c) The molecular function of GO analysis. (d) KEGG pathway.

To explore the underlying mechanism of ferroptosis in psoriasis, we used the online tool Metascape to analyze the GO and KEGG of 11 differential genes, shown in the chord diagram (Figure 4). Enriched biological processes in the GO analysis included cellular response to chemical stress, response to nutrient levels, carboxylic acid transmembrane transport, and wound healing (Figure 4(a)). The cell component terms of GO analysis included apical part of the cell and apical plasma membrane (Figure 4(b)). The molecular functional terms of GO analysis were organic anion transmembrane transporter activity, protein homogenization activity, and kinase binding (Figure 4(c)). With KEGG pathway analysis, DEGs were mainly rich in central carbon metabolism in cancer and HIF-1 signaling pathway (Figure 4(d)).

### 3.5. Validation of Gene Expression Associated with Ferroptosis in the Other Dataset

**Figure 5:**
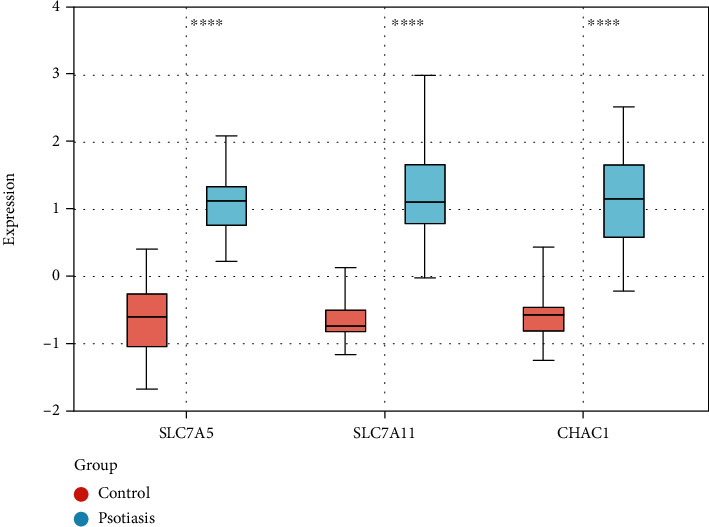
Validation of gene expression in the GSE13355 dataset. Box plots showing 3 differentially expressed ferroptosis-related genes in skin tissues of patients with psoriasis and healthy people. The Wilcox test was used.. ^∗∗∗∗^- *P* value <0.0001 compared to control.

To verify the reliability of these 11 ferroptosis-related gene expression levels, we selected the GSE13355 dataset containing skin tissue from 58 patients with psoriasis and 64 normal health controls [19]. Similar to the results of paired samples in the GSE30999 dataset, the expression levels of SLC7A5, SLC7A11, and CHAC1 were increased in the psoriasis lesions (1.85-, 1.73- and 1.70-fold, respectively) compared to normal skin tissues. However, the expression levels of MUC1, GPT2, G6PD, SRXN1, STAT3, HMOX1, SLC7A1, and MAPK14 did not differ significantly between the two groups (Table 3 and Figure 5).

### 3.6. Prediction of Potential Therapeutic Drugs

The DGIbd and CMAP databases were used to find potential drugs for these three genes mentioned above. In the DGIbd database, Melphalan was found to be the targeted medicine of SLC7A5, and Quisqualate or Riluzole was found to be the targeted medicine of SLC7A11. While two drugs, including Riluzole and Sulfasalazine, were found in the CMAP database (Table 4). CHAC1 was included neither in DGIbd nor CMAP.

### 3.7. Molecular Docking Analysis

**Figure 6:**
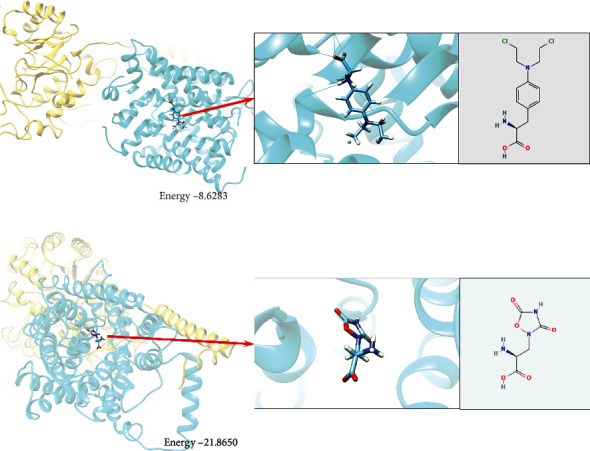
Molecular docking simulation. (a) Left: Melphalan-SLC7A5, Energy = −8.6283. Right: Chemical formula of Melphalan. (b) Left: Quisqualate-SLC7A11, Energy = −21.865. Right: Chemical formula of Quisqualate.

The chemical structure of Melphalan, Quisqualate, Riluzole, and Sulfasalazine was acquired from the ZINC database for molecular docking analysis. Subsequently, two target proteins were examined from the RCSB PDB database, including SLC7A5 (PDB ID: 7DSL) and SLC7A11 (PDB ID: 7EPZ). Finally, molecular docking was carried out using the SwissDock tool. Molecular docking results showed that all binding energies were negative. On the other hand, results showed that the combination of Melphalan-SLC7A5 and Quisqualate-SLC7A11 was the most stable (Figure 6(a) and 6(b)). The docking ligand-protein binding energy and fullfitness energy are summarized in Table 5.

## 4. Discussion

Ferroptosis is a recently discovered form of programmed cell death, which relies on iron-driven lipid peroxidation [24, 25]. On the one hand, reducing iron levels is one of the therapeutic strategies for treating hemochromatosis and acute lung injury [26, 27]. On the other hand, inducing ferroptosis might be an effective strategy for killing tumor cells and reducing liver fibrosis [28, 29]. While ferroptosis has been implicated in many diseases, the relation between ferroptosis and skin pathophysiology remains largely unexplored. A recent study explored the correlation between psoriasis and ferroptosis. The study examined the tendency of ferroptosis in clinical samples and erastin-treated human primary keratinocytes, the Imiquimod (IMQ)-induced model of psoriasis [10]. Their results showed that some specific molecules of ferroptosis (PTGS2, 4-HNE, and ACSl4) enhanced the inflammatory response of psoriasis. However, the expression of key regulators in ferroptosis (SLC7A11and GPX4) suggested that ferroptosis was inhibited in psoriasis, which was not explained in this study. More data are needed to analyze the expression of ferroptosis genes in psoriasis. A better understanding of the underlying mechanisms of ferroptosis and psoriasis may accelerate the development of promising treatment strategies. Therefore, we aimed to identify potential ferroptosis-associated genes in psoriasis by bioinformatics analysis. We analyzed the intersection of DEGs in the GEO database (GSE30999 dataset), validated the gene set of ferroptosis in the FerrDb database, and obtained 19 genes.

Subsequently, 11 genes constituting the molecular network in the PPI network were screened. Functional analysis of ferroptosis-associated genes showed that these genes were related to the central carbon metabolism in cancer and the HIF-1 signaling pathway. Changes in carbon metabolism in cancer centers, including aerobic glycolysis, elevated glutaminolysis, imbalance of tricarboxylic acid cycle, and changes in the pentose phosphate pathway, promote cancer development by maintaining viability and building new biomass for cancer cells [30]. The HIF-1 signal pathway is considered a classical pathway related to various oxidative stress responses, but few studies have investigated the relationship between HIF activation and ferroptosis. Recent studies showed that the silenced HIF-1*α* could reduce the level of SLC7A11 protein, whereas plasmid or stabilizer overexpressing HIF-1*α* could increase the level of SLC7A11 protein. The reduction of HIF-1*α* and SLC7A11 in Hepatic stellate cells (HSC) enhanced sorafenib-induced cell ferroptosis and extracellular matrix (ECM) reduction. Conversely, increased expressions of HIF-1*α* and SLC7A11 inhibited HSC ferroptosis and impaired the sorafenib antifibrotic effect [31, 32]. Therefore, suppressing the HIF-1*α*/SLC7A11 pathway could induce HSC ferroptosis.

Based on the above bioinformatics analysis, the expression levels of 11 differentially expressed ferroptosis-related genes were further evaluated with the GSE13355 dataset. As a result, the expression levels of SLC7A5, SLC7A11, and CHAC1 were upregulated, consistent with that of the GSE30999 dataset. Both SLC7A11 and SLC7A5 are members of the heteromeric amino acid transporter group. As a member of the solute transport family, SLC7A11 encodes a cystine/glutamate xCT transporter, a crucial protein regulating iron overload-ferroptosis and could be reduced to cysteine for GSH synthesis [33]. It was discovered that pharmacologic blockade of SLC7A11-mediated cystine uptake by compounds (e.g. with erastin, sulfasalazine, or sorafenib) induces ferroptosis [4]. Interestingly, in our study, the expression of SLC7A11 mRNA in psoriasis lesions was upregulated, in accordance with a previous report on ferroptosis in psoriasis [10]. However, no direct evidence exists that SLC7A11 is related to psoriasis by mediating ferroptosis. There are similarities in pathology and therapeutic targets between psoriasis, tumor, and liver fibrosis; therefore, we referred to the recent relevant reports. Many reports have shown that SLC7A11-mediated regulation of ferroptosis plays a crucial role in cancers, while several cancer immunotherapy methods that are also effective in treating psoriasis show the significance of inhibiting SLC7A11 and inducing ferroptosis in tumor cells [29]. For example, Bavarian, which has an antitumor effect, promotes ferroptosis in osteosarcoma (OS) cells by inhibiting the STAT3/p53/SLC7A11 axis [34]. Trim26 promotes HSC ferroptosis in liver fibrosis by mediating SLC7A11 ubiquitination to inhibit liver fibrosis, which may be a new treatment strategy [28]. Moreover, the knockout of SLC7A11 in normal cells does not induce ferroptosis, suggesting that targeting SLC7A11 might be safe in clinical treatment [26]. Similar to psoriasis, abnormal cell proliferation is the key pathological feature of liver fibrosis. We speculate that abnormal proliferation of keratinocytes is the main pathological feature in psoriasis; therefore, inducing ferroptosis of keratinocytes might be a new therapeutic strategy for psoriasis. Although recent reports support some of our conjectures, the role of SLC7A11 in regulating psoriasis and ferroptosis needs further study.

SLC7A5 is an important L-type amino acid transporter 1 (LAT1) to facilitate the uptake of its substrate leucine. In many cancerous tissues and some skin diseases, SLC7A5 is overexpressed [35]. By using psoriasis mouse models, induced by imiquimod (IMQ) and IL-23, Danay Cibrian found that targeting LAT1-mediated amino acid uptake is a potentially useful immunosuppressive strategy to control skin inflammation mediated by the IL-23/IL-1*β*/IL-17 axis [36]. In addition, similar to SLC7A11, SLC7A5 has a quite similar regulation of factors involved in ferroptosis signaling. The expression of SLC7A5 and SLC7A11 was reported to be increased by sublethal concentrations of ferroptosis inducers, which could help cells to cope with oxidative stress [37].

CHAC1 encodes a proapoptotic protein with glutathione-specific *γ*-glutamyl cyclotransferase activity and induces GSH degradation [38]. The upregulation of CHAC1 is widely accepted as an early ferroptotic marker [39]. Interestingly, recent research has revealed that CHAC1, like SLC7A11, is downregulated by the loss of YAP/TAZ, inducing ferroptosis [40]. In psoriasis, YAP signaling is activated by IL-17A, promoting keratinocyte proliferation [41]. However, the role of CHAC1 in the execution of ferroptosis and its involvement in psoriasis is unclear.

Finally, molecular docking identified several potential therapeutics related to ferroptosis-related genes for psoriasis, including Melphalan, Quisqualate, Riluzole, and Sulfasalazine. Melphalan is a common chemotherapeutic drug reported to have therapeutic effects on severe psoriasis. Paclitaxel is a chemotherapeutic drug with antiproliferation, antiangiogenesis, and anti-inflammatory properties. Micellar paclitaxel shows therapeutic activity in patients with severe psoriasis, with most patients showing a good tolerance to the drug [42]. However, as an important chemotherapeutic drug for the treatment of malignant tumors and immunoglobulin light chain amyloidosis (AL amyloidosis) [43, 44], very few studies have reported the treatment of psoriasis by Melphalan. Chen reported a rare case of concurrent AL amyloidosis and psoriasis. It was found that the chemotherapeutic regimen based on bortezomib and thalidomide achieved partial hematological remission, but the kidneys were unresponsive, and psoriasis was still active. However, after receiving intravenous Melphalan and hematopoietic stem cell transplantation (HSCT), the patient achieved complete hematological remission, organ response, and the disappearance of psoriasis. These results indicate that Melphalan as a chemotherapeutic drug has potential therapeutic effects on AL amyloidosis combined with psoriasis. Sulfasalazine is a conventional synthetic disease-modifying antirheumatic drugs (csDMARDs), which has been recognized as the first-line treatment for psoriatic arthritis (PsA) [45]. However, there is no information about using Quisquamate to treat specific diseases in the Drugbank database. Also, the relationship between Riluzole and psoriasis treatment is unclear.

A few limitations also existed in our study. Firstly, the sample size in this study was relatively small, and the genes related to ferroptosis may be incomplete. In addition, the study is based on data analysis. Therefore, more microarray data and biological experiments are needed to verify the results by analyzing IC50, gene mRNA expression, and protein expression.

## 5. Conclusion

Thus, our results indicate SLC7A5, SLC7A11, and CHAC1 as the underlying biomarkers for psoriasis, providing further evidence about the crucial role of ferroptosis in psoriasis.

## Figures and Tables

**Table 1:** Baseline demographic characteristics of 89 psoriasis patients with skin lesion and no-lesion biopsy specimens.

Information	Psoriasis patients (*n* = 89)
Age (years)	44.6 ± 13.1
Male	66 (77.5%)
Caucasian	75 (84.3%)
Current smoker	36 (40.4%)
Obese	47 (52.8%)
Body surface area with psoriasis (%)	30 ± 20.5
Psoriasis area and severity index score	21.5 ± 10.8
Psoriatic arthritis	19 (21.3%)

Data shown are the number (%) of patients or mean ± standard deviation.

**Table 2:** Differential expressions of ferroptosis-associated genes.

Gene	logFC	Change	*P* value	Adj. *P* value
MUC1	-2.61635294	Down	9.85E-13	7.17E-12
PLIN4	-2.60952941	Down	1.38E-11	8.82E-11
HBA1	-1.88611765	Down	4.35E-06	1.55E-05
GPT2	1.6808235	Up	3.38E-32	1.83E-30
PANX1	1.7144706	Up	1.03E-28	3.79E-27
G6PD	1.7154118	Up	1.10E-20	1.81E-19
SRXN1	1.7883529	Up	7.63E-28	2.55E-26
STAT3	1.8606471	Up	1.47E-25	3.85E-24
SLC7A5	1.8842353	Up	2.05E-31	1.02E-29
BID	1.8962353	Up	6.18E-23	1.29E-21
HMOX1	1.9029412	Up	6.89E-20	1.04E-18
SLC7A11	2.0696471	Up	2.54E-28	8.91E-27
SLC2A1	2.2173529	Up	4.24E-35	3.10E-33
MIR17	2.3104706	Up	6.58E-22	1.23E-20
ALOX12B	2.3361176	Up	7.23E-32	3.78E-30
MAPK14	2.6892353	Up	4.49E-46	9.43E-44
AURKA	3.0157647	Up	1.41E-30	6.48E-29
RRM2	3.3896471	Up	1.24E-50	4.49E-48
CHAC1	4.8998824	Up	2.22E-46	4.97E-44

**Table 3:** Validation of ferroptosis-related genes expression in the GSE13355 dataset.

Gene	logFC	Change	*P* value	Adj. *P* value
SLC7A11	1.8590156	Up	7.74E-43	8.55E-41
SLC7A5	1.7331389	Up	2.85E-40	2.31E-38
CHAC1	1.7025686	Up	3.75E-36	1.95E-34

**Table 4:** Prediction of potential therapeutic drugs.

Compound	Gene	Type	Source	PubChem ID	Citation
Melphalan	SLC7A5	Inhibitor	DGIbd	460612	[20]
Quisqualate	SLC7A11	Inhibitor	DGIbd	40539	[21]
Riluzole	SLC7A11	Inhibitor	CMAP/DGIbd	5070	[22]
Sulfasalazine	SLC7A11	Inhibitor	CMAP	5359476	[23]

**Table 5:** Molecular docking analysis.

Target	Compound	FullFitness (kcal/Mol)	Energy (kcal/Mol)
SLC7A5	Melphalan	-3388.5396	-8.6283
SLC7A11	Quisqualate	-3866.7695	-21.8650
	Riluzole	-3841.4240	-6.9729
	Sulfasalazine	-3892.8599	-0.5501

## Data Availability

The datasets generated and/or analyzed during the current study are available in the [GSE30999] repository, [https://www.ncbi.nlm.nih.gov/geo/query/acc.cgi?acc=GSE30999]; in [GSE13355] repository, [https://www.ncbi.nlm.nih.gov/geo/query/acc.cgi?acc=GSE13355].
